# Oxygen Sensor-Based Respirometry and the Landscape of Microbial Testing Methods as Applicable to Food and Beverage Matrices

**DOI:** 10.3390/s23094519

**Published:** 2023-05-06

**Authors:** Dmitri B. Papkovsky, Joseph P. Kerry

**Affiliations:** 1 School of Biochemistry and Cell Biology, University College Cork, Pharmacy Building, College Road, T12 YT20 Cork, Ireland; 2 School of Food and Nutritional Sciences, University College Cork, Microbiology Building, College Road, T12 YT20 Cork, Ireland

**Keywords:** microbial testing, rapid methods, food quality and safety, optical oxygen respirometry, oxygen sensor and biosensor systems

## Abstract

The current status of microbiological testing methods for the determination of viable bacteria in complex sample matrices, such as food samples, is the focus of this review. Established methods for the enumeration of microorganisms, particularly, the ‘gold standard’ agar plating method for the determination of total aerobic viable counts (TVC), bioluminescent detection of total ATP, selective molecular methods (immunoassays, DNA/RNA amplification, sequencing) and instrumental methods (flow cytometry, Raman spectroscopy, mass spectrometry, calorimetry), are analyzed and compared with emerging oxygen sensor-based respirometry techniques. The basic principles of optical O_2_ sensing and respirometry and the primary materials, detection modes and assay formats employed are described. The existing platforms for bacterial cell respirometry are then described, and examples of particular assays are provided, including the use of rapid TVC tests of food samples and swabs, the toxicological screening and profiling of cells and antimicrobial sterility testing. Overall, O_2_ sensor-based respirometry and TVC assays have high application potential in the food industry and related areas. They detect viable bacteria via their growth and respiration; the assay is fast (time to result is 2–8 h and dependent on TVC load), operates with complex samples (crude homogenates of food samples) in a simple mix-and-measure format, has low set-up and instrumentation costs and is inexpensive and portable.

## 1. Introduction

Approximately 25% of global food loss is due to microbial spoilage, which incurs significant economic and environmental burdens for producers [1]. Spoilage is the process of food deterioration leading to differences in appearance (compared to fresh), development of an off-odor and changes in texture and color [2]. Initial microbial colonization can come from soil, personnel, water, air and equipment used, which are the biotic factors. The selection and subsequent dominance of certain bacteria are influenced by abiotic factors, such as a gaseous atmosphere, pH, temperature and salinity levels [2]. The primary bacterial entities associated with meat spoilage, for example, are *Enterobacteriaceae*, *Pseudomonas*, *Brochothrix thermosphacta* and *Lactobacillus* spp. [2,3]. Alongside microbial spoilage, bacteria such as *Escherichia coli* strains, *Staphylococcus aureus*, *Listeria monocytogenes* and *Salmonella* spp. cause foodborne diseases, which have become a widespread concern for productivity losses and public health [3].

Fresh meat products, similar to many fresh food products, are highly susceptible to microbial contamination due to minimal pre-treatment and richness of moisture and essential nutrients [3]. To monitor spoilage and pathogenic bacteria, the meat industry, for example, utilizes meat samples and swabs for surface hygiene assessments. Food samples are usually subjected to homogenization with a recovery diluent and stomacher-producing crude homogenates, which are then analyzed. This technique allows for an almost complete recovery of viable bacteria from the product [4]. For the swabs, a cotton brush or sponge with a recovery diluent is used to sample a food/non-food surface and create a bacterial suspension. Although the recovery rate is not as high as with food samples [4], swabbing allows for the tracing of contamination and cross-contamination in environmental and microbiological surveillance [5]. 

## 2. Culture-Based Methods of Microbiological Testing

The ‘gold standard’ colony count methods ISO 4833-1:2013 [6] and ISO 18593:2018 [7] adopted widely by the industry and microbiological labs determine the total aerobic viable counts of bacteria (TVC) in food, environmental or swab samples. This is performed by taking 10 g samples, homogenizing them in 90 mL of the MRD (maximal recovery diluent), making 1:10 serial dilutions of the homogenates, and applying them onto solid growth media in agar plates. The plates are incubated at an optimal temperature (from 7 °C to 55 °C) and atmosphere (aerobic) for the target microorganism, typically for 24–72 h. Each viable cell (called a colony forming unit, CFU) produces a colony; such colonies are counted and used to calculate the TVC number for the original sample/product. The limit of detection for this method is approximately 4 CFU/mL for liquid foods and 40 CFU/g for solid foods [8]. 

If a sample is expected to have low TVC (<50 CFU/g), the most probable number (MPN) method is used in which 3 serial dilutions of the sample are made, then each dilution is dispensed into 9 or 15 tubes, and the number of positive tubes for each dilution is counted [8]. Due to the high level of uncertainty, the MPN method is used solely for estimation. Both methods are considered labor-intensive and time-consuming, as they require laboratory expertise, large amounts of consumables and lengthy incubation periods. 

Besides the non-selective growth media, plates with selective media containing special additives, which promote the growth of target bacterial species while suppressing all other microbiota, are also used. Thus, bile salts, ox gall and methylene blue and crystal violet dyes can inhibit Gram-positive bacteria while allowing Gram-negative to grow. In addition, Gram-negative bacteria are inhibited by LiCl, NaN_3_ or acriflavine, which promote the growth of Gram-positive bacteria [9]. Chromogenic and fluorogenic substrates and additives are also used to detect microorganisms with the corresponding enzymes and metabolic systems [8]. TVC assays on selective media also allow for predictive identification of particular bacterial species and microbial pathogens, such as Listeria, Salmonella, etc. 

Modern versions of the above colony counting methods are compact, pre-made plates, such as 3 M Petrifilm, Compact Dry, and SimPlate, which contain dehydrated nutrients and differentiating components on dedicated films or plates that aid in enumeration [8]. Such plates are easy to use and are space- and labor-saving but still require special usage expertise, sample preparation time and a delayed period in ascertaining results.

### 2.1. The TEMPO System 

The more advanced and automated version of traditional culture-based TVC methods is represented by the TEMPO system developed by bioMerieux [10,11]. TEMPO uses dedicated, small-size plastic cassettes with an array of fluidic channels. Each channel contains a sampling port, small reservoirs pre-loaded with dry media and a special fluorescent dye and a measurement chamber with transparent windows used for fluorescent detection of the dye signal. Tested samples homogenized in a stomacher (if required) are loaded into pre-existing channels with a special device, and then the cassette is moved to an incubator where it is left for several hours or overnight. Following incubation, the cassette containing samples is brought back and read on an automatic fluorescent reader which produces a result for each sample [11]. Although faster, less laborious and more automated than the ISO 4833-1:2013 method, the TEMPO system is not used routinely with classical TVC assays due to the high costs associated with equipment and assays. 

Conversely, the TEMPO system is actively used for the predictive identification of specific pathogens using the cassettes pre-loaded with dehydrated selective media for specific pathogen detection (also developed by bioMerieux [12]). The test may include a pre-enrichment step in a selective liquid medium; it often produces a qualitative or semi-quantitative output (below or above the set CFU/g threshold) and requires confirmation by selective molecular methods. 

### 2.2. The Soleris System

The Soleris/Neogen system operates with liquid growth media, disposable, specially profiled assay vials and colorimetric CO_2_ sensor inserts. For the assay, samples are homogenized, diluted with growth media and dispensed into the vials. Sensor film inserts are then inserted into each vial, which is then capped, placed in an incubator and monitored visually. Once the bacteria in the vial reach a certain density and metabolic activity, they produce a sufficient quantity of CO_2_, which is detected by the sensor insert via color change [13]. The time of the color change is related to the TVC of the sample. Visual detection of color changes provides qualitative estimates of the TVC (present/not present, above/below the given threshold), while an instrumental colorimetric readout gives semi-quantitative results. The system shares similarities with O_2_ respirometry but is less robust and accurate since absorbance measurements are more influenced by the samples matrix. Nevertheless, it is actively used in selective TVC assays for the predictive identification of microbial pathogens [14]. 

## 3. Bioluminescent Detection of Cellular ATP

Living cells contain ATP, which is essential for the maintenance of enzyme systems, biosynthesis of cellular components and regulation of stored metabolic energy [15]. Being one of the key metabolites and the main energy currency of the cell makes ATP a useful marker of cell viability [16], and the bioluminescent (BL) reaction between luciferin and luciferase enzyme can be used for ATP quantification. This reaction occurs at low ATP levels; the amount of emitted light is proportional to the ATP concentration and can be measured on simple luminometers and expressed in relative light units (RLU) [8,17]. However, the ATP assay requires cell lysis and the release of cellular ATP. It is also susceptible to quenching of BL by sample matrix, provides only a rough estimate of bioload present and cannot differentiate between microorganisms. Additionally, food samples, such as meat or milk, can contain somatic cell ATP, which can cause interference and high background levels of BL. In order to detect microbial ATP, pre-treatment of samples is required [15,18]. Subsequently, to convert RLU signals into CFU/g, a standard curve must be established using known standards of bacteria [15]. 

Ready-to-use BL kits for total cellular ATP levels with portable luminometers are commonly used to monitor surface hygiene [15,17]. Thus, ready-to-use, pre-moistened surface swabs from 3 M (Clean-Trace Surface ATP Test Swab UXL100) were used to evaluate bacterial populations on supermarket food contact surfaces. Interestingly, surfaces that were deemed satisfactory in traditional plate count tests were in fact deemed unsatisfactory based on ATP BL assessment. This difference could be accounted for by the presence of biofilms (which would be underestimated by traditional methods) or viable but non-culturable cells (VBNC) which contain similar ATP levels to viable cells [17]. The samples used were relatively clean and therefore did not require additional treatment apart from cell lysis.

The effects of various interfering compounds associated with fish processing on ATP BL were investigated with two commercial kits [19]. NaCl and artificial acidic liquid smoke additives had the highest rates of BL quenching and RLU values of the same sample varied between kits. Although based on the same principle, the results from these kits were not directly comparable and must be converted using a standard curve. High-protein and high-fat fish samples also showed large variations in ATP BL, as the cells proved to be difficult to lyse. The different stages of fish processing and possibly other food processing plants would therefore require different acceptable levels of RLUs to ensure proper hygiene and subsequent food safety [19]. With the increase in the complexity of samples, there is a decrease in ATP BL, and additional sample pre-treatment is required, which is not provided by most of the available kits. 

ATP BL was also applied to the detection of yeast and bacteria in wine; however, special treatment of samples was required. Wine samples were filtered through 2 sets of membrane filters, which were then incubated in selective media for 24 h with added ATPase (hydrolyses free ATP) and analyzed via luminometer to eventually discern between varying levels of contamination with yeast and bacteria [18]. 

## 4. Molecular Methods

The introduction of selective molecular methods that focus either on protein/antigen markers or the DNA/RNA of target pathogens provided alternatives and complementary techniques to traditional microbiological methods. While requiring a higher level of laboratory expertise, these techniques offer a higher specificity than traditional methods, with selective detection of particular bacterial species and strains in complex mixtures. Since these techniques do not provide single-cell sensitivity, they often include a ‘pre-enrichment’ step by culturing test samples on selective media. 

### 4.1. Immunoassays 

Immunoassays utilize the highly specific recognition and binding of various antigens by corresponding antibodies that can be raised in animals or produced by hybridoma technologies. They are widely used in food diagnostics as enzyme-linked immunosorbent assay (ELISA) kits in which an immunoassay is coupled with a dedicated solid substrate and an enzyme label [8,20]. Bacterial cells are normally quantified by sandwich ELISAs with a pair of antibodies specific to cell surface markers. The first antibody is adsorbed on a plastic surface and used to capture the cells of interest from the sample. Then, the second antibody labeled with an enzyme binds to this complex and generates a signal proportional to the number of cells. The multistep ELISA process (coating, blocking, specific binding, washing and signal generation) is labor-intensive and time-consuming and can be interfered with by many factors, including sample matrix components, pH, ions, salt, temperature, etc. [21]. The extraction of desired protein markers for immunoassays from complex homogenates is often required, which further complicates the assay [22,23]. Furthermore, the test cannot distinguish between viable and non-viable cells. 

#### 4.1.1. Modifications of ELISA Tests

The classical ELISA format has numerous modifications in general and in microbial testing in particular. Examples include immunochromatography tests on filter paper (Whatman No.1) for the detection of *Escherichia coli* 0157: H7, which took <3 h, required only 5 μL of sample and had a detection limit of 10^4^ CFU/mL [24]. For visualization of results via tetramethylbenzidine-hydrogen peroxide (TMB-H_2_O_2_) reaction, a scanner or smartphone and Image J software can be used. Another modification of an ELISA used cotton swabs both as cell collectors and detection devices. Bacterium-specific antibodies were immobilized on cotton swabs, which were then submerged in a colored nanobead-antibody conjugate solution to create a sandwich ELISA with an LOD of 10 CFU/mL. Semi-quantitative results were produced from the color changes on the swabs corresponding to specific bacteria, which could also be quantified with a smartphone and Image J [25]. As only artificially contaminated surfaces were tested, the cotton swabs may be limited by potentially complex contamination present in industrial settings. Furthermore, the stability of the cotton swabs was not tested and could provide limitations for production and storage. 

#### 4.1.2. Lateral Flow Assays

Another common modification of ELISAs, used, e.g., in pregnancy and COVID-19 antigen testing, is called lateral flow assays (LFA). LFAs are also available for the testing and quantification of various microbial pathogens [26]. Such LFA devices utilize different internal architecture, materials and fluidic networks, labels and detection systems [27] but still operate in a similar manner: take a sample, apply it on the device, wait for the immune reaction to occur and read the result. These simple and inexpensive point-of-care devices do not require complex procedures and sample preparation steps, and they provide good (though not ultimate) sensitivity and fast, qualitative visual results without any special equipment. Their sensitivity is moderate and comparable with cell-based ELISAs. 

#### 4.1.3. Immuno-Magnetic Separation Systems (IMS) 

IMS systems usually operate with dispersions of magnetic microbeads coated with specific antibodies that can selectively recognize and tightly bind to their targets, including microbial cells. When such microbeads are added to the sample, they quickly capture target cells. Subsequently, by means of a special magnet, the beads can be pulled out from the sample to the wall of the assay vial, thus separating the cells from the rest of the sample which can be discarded [28,29]. So, the microbeads and IMS effectively extract and concentrate the cells of interest, which then can be (i) cultured in appropriate growth media and quantified by plate counting method (TVC counts) or (ii) detected on beads by a sandwich ELISA using a labeled antibody that produces a signal and quantitative readout. So, IMS techniques combine the benefits of ELISAs and culturing methods. The magnetic separation/enrichment step with solution-based binding reaction is fast, simple and efficient; it boosts the detection sensitivity of the subsequent detection by ELISA [30] or by direct plating [31]. However, IMS assays are costly and labor-intensive, and their ELISA version does not ensure the detection of only viable cells (as the culturing version does). 

### 4.2. Polymerase Chain Reaction (PCR) Methods 

Another group of common molecular techniques in the detection of bacteria in food relies on the isolation of bacterial RNA, its reverse transcription into cDNA and the amplification of cDNA, mainly by the polymerase chain reaction (RT-PCR). PCR uses a DNA polymerase system to copy a specific region on a template strand of DNA defined by a choice of short oligonucleotide primers that have matching sequences to the end region of interest. Amplification occurs over a series of cycles in which the sample is heated and cooled to produce double-stranded DNA of interest [8]. Using special hybridization probes recognizing the target sequence, a real-time or quantitative PCR (qPCR) system can also be set up, which combines cyclic amplification and fluorescent detection and quantification of the target DNA [8]. Multiplex PCR with multiple sets of primers and probes allows for the simultaneous detection of multiple targets in a single reaction tube [8,32].

Thus, a qPCR screening method for Salmonella in environmental swabs was developed with primers and probes based on the gene invA, group D and *Salmonella enterica serovar Enteritidis*. Its high specificity was demonstrated on a panel of 329 *Salmonella* isolates with results produced in 18–24 h. Sample pre-enrichment was still necessary for the described assay [33]. Another qPCR assay was developed for the detection of *Staphylococcus* spp. in meat products using specific primers to the conserved regions of enterotoxin genes. The assay time was 12 h including an 8 h pre-enrichment step, and the LOD was 2–40 CFU/g [34].

### 4.3. Loop-Mediated Isothermal Amplification (LAMP)

Loop-mediated isothermal amplification (LAMP) was developed as a simpler alternative to PCR without thermal cycling. LAMP uses a set of four special primers that recognize a total of six distinct regions within the target sequence of DNA. The inner primer with both sense and antisense strands initiates LAMP while the outer primer allows for strand displacement, releasing single-stranded DNA. This strand of DNA then acts as a template primed by the second inner and outer primers which produce stem-looped DNA. In subsequent cycles, one inner primer hybridizes into the loop and initiates displacement DNA synthesis. The reaction lasts 1 h at 65 °C and produces 10^9^ copies of DNA, which include stem-loop and cauliflower-like structures with multiple loops [35].

A multiplex real-time LAMP assay was established to simultaneously detect and differentiate *Salmonella* spp. and *Vibrio parahaemolyticus* DNA within a single tube. The amplified products were then subjected to melting curve analysis, which clearly showed a distinction between the products. The assay showed 100% inclusivity and exclusivity and sensitivity similar to that of multiplex PCR [36]. Another multiplex LAMP combined with a lateral flow dipstick was developed for the rapid detection of *Salmonella* spp., *Carnobacter* spp. and *Staphylococcus aureus* in powdered infant formula. The accumulation of sandwich composites formed a red band on the device, allowing for visual inspection. The LODs for *Salmonella* spp., *Carnobacter* spp. and *S. aureus* without enrichment were 4.2, 2.6 and 3.4 log(CFU/g), respectively, with the entire assay completed within 1 h [37].

Although LAMP does not require expensive laboratory equipment, it still necessitates sophisticated DNA extraction procedures which are performed by skilled personnel. Moreover, complex heterogeneous food samples could affect DNA isolation and cause contamination.

### 4.4. Next-Generationation Sequencing Methods

While the aforementioned molecular methods target just one or several specific markers of bacterial species, with next-generation sequencing (NGS), whole microbial communities can be examined in greater detail for their functional diversities. The main categories of NGS include (i) amplicon sequencing of specific marker gene families and (ii) metagenomics with random shotgun sequencing for the whole genomic content of communities [38]. Both sequencing types require the purification and isolation of DNA from samples. With amplicon sequencing, the extracted DNA undergoes targeted PCR amplification, commonly the 16 S rRNA marker gene for bacteria [38]. Indices are then attached to these amplicons by an additional round of PCR. The resulting DNA libraries are normalized, pooled and sequenced. This allows for the identification of the microbial community at a genus level and can follow the succession of microbial populations over time [38]. In metagenomics, the extracted DNA molecules undergo fragmentation followed by shotgun sequencing. Reads are then classified into various genomic locations. This in turn allows for the analysis of individual strains and the prediction of their function within the microbial community [38]. Both sequencing types produce large datasets which are processed with bioinformatics pipelines. 

Metagenomic sequencing was used to analyze differences in chicken breast microbiomes and profile antimicrobial resistance genes. It revealed a greater effect of packaging type and processing environment on microbiome composition than antibiotic usage and seasonality. Furthermore, the composition of the poultry microbiomes could be indicative of potential metagenomic markers for food safety and quality, which in turn could be translated to the evaluation of processing environments and practices [39].

A combination of amplicon and target-enriched shotgun sequencing was used to profile the microbiome and resistome of ground beef products in the US. The results indicated no difference between products that claimed ‘raised without antibiotics’ and those raised by conventional means. Similar to the above, this study found that product management had a greater influence on the ground beef microbiome than the resistome. Furthermore, the novel target-enriched shotgun sequencing implemented in this study allowed for the analysis of samples with low microbial abundance [40]. 

Although NGS has many advantages and a high potential for food diagnostics and safety assessment, there are also limitations. The sample preparation and subsequent analysis are complicated, labor-intensive and expensive. The analysis of the data produced requires skilled experts and is time-consuming. Furthermore, low microbial abundance and high amounts of host (off-target) DNA can limit the effectiveness of NGS techniques.

For all the above RNA/DNA detection and amplification techniques, sophisticated isolation and purification of the nucleic acid fractions from food samples is necessary. The food matrix is complex and heterogenous; its particulate matter, biochemical compounds and indigenous microbiota can interfere and inhibit PCR assays [34,41], which are also susceptible to contamination and require highly specific primers for each target.

## 5. Instrumental Methods

Instrumental techniques have been widely applied to the analysis of food samples and the assessment of their microbial safety. While fulfilling similar general analytical tasks, their measurement methodologies, detection platforms and underlying principles vary greatly. The main methods which have found wider use in the microbiological assessment of food samples include the following. 

### 5.1. Flow Cytometry and Cell Counting 

Flow cytometry (FCM) measures the optical characteristics of individual cells when they pass one by one in a flow channel through a focused laser beam [8]. For each particle, the following optical signals are measured: forward scatter, side scatter and fluorescence. The forward and side scatter are determined by cell size and granularity (i.e., internal structures) and can be used for the identification of cells. Additional fluorescent staining with labeled antibodies or dyes that can report on the metabolic state and viability of microorganisms largely improves method selectivity and allows one to distinguish between debris, viable and dead cells and different types and sub-classes of microorganisms [8]. Being able to rapidly separate, count and group individual cells on the basis of several measured parameters, the analytical power and utility of flow cytometry is very high. However, it cannot operate with crude and complex samples containing particulate matter (e.g., food homogenates). 

For example, a staining system combining fluorescently labeled antibodies and propidium iodide (cell-permeable red fluorescent dye) was developed to detect viable *S. aureus* cells in milk and milk powder using FCM. With a 5 h pre-enrichment period, the method could detect low numbers of *S. aureus* in 6 h with an LOD of 7.50 cells/mL for milk and 8.30 cells/g for milk powder [42]. Another FCM method provided the detection and differentiation of probiotic strains of *B. subtilis* and other *Bacillus* species. Using the two cell-permeant fluorescent stains: green nuclear stain, SYTO24 and Laser Dyes Styryl 751, FCM could differentiate three subpopulations: spores, vegetative cells and VBNC/ dead cells [43]. 

FCM was also used to quantify the effects of electrolyzed water on *E. coli* O157:H7 and *Listeria monocytogenes*. Using the cell-permeable green fluorescence stain SYTO^®^ 9 and propidium iodide, it was shown that at low chlorine concentrations in electrolyzed water, cells were induced into a VBNC state as a significant portion of cells retained cell integrity and emitted green fluorescence [44]. 

In FCM analysis, the sample itself is a limiting factor: heterogeneous and particulate samples, such as food homogenates, are not suitable for FCM or interfere with readings by producing high background noise. Fluorescent staining of samples also requires optimization, skilled personnel and a laboratory setting. FCM equipment is expensive and requires special skills to operate it. 

### 5.2. Raman Spectroscopy 

Raman spectroscopy uses the effect that laser light can be scattered non-elastically when interacting with a sample via energy transfer between incident photons and sample molecules [45]. The amount of energy transferred is small and directly corresponds to specific vibrations within a molecule, which can be visualized in unique Raman spectra. However, Raman spectra can be masked by a broad and intense optical background, and it requires sample preparation that preserves intact bacterial cells [45]. 

Single-cell Raman spectra (SCRS) can be generated using the same principle and irradiating a single bacterial cell. Different vibration modes of various chemicals and cell components (proteins, polysaccharides, NAs, lipids) of a given bacterial cell are reflected in SCRS and provide a unique Raman signature for it without the need for any pre-treatment or pre-incubation [46]. Although highly specific, SCRS are difficult to classify by eye, as different bacterial cells have minute differences in spectra. To overcome this, databases of Raman spectra are produced and analyzed by chemometrics. Depending on the application and analytical task, a classification model is produced via machine learning and then validated with independent spectra not included in the original database. The database can then be used for the identification of unknown samples [45]. Compared to genotypic methods, such as NGS, Raman spectroscopy is a relatively rapid and inexpensive method for the classification of bacterial species [47].

Thus, a database of SCRS from seven common food-borne pathogen genera (23 strains) was created, and chemometrics was applied for its analysis. Machine learning helped distinguish SCRS and achieved the bacterial classification of 87.1–95.8% at the serotype level [46]. This dataset was suggested for the identification of food-borne pathogens in food samples; however, this has yet to be tested. SCRS was also applied to classify six food-spoilage bacteria and investigate their tolerance to food additives. Using vector machine learning, Raman spectra were able to distinguish bacterial species and three types of stress conditions with different classes within each type based on minimum inhibitory concentrations (MIC). The model was able to classify species with 88.2% accuracy and stress tolerances with 91.2% accuracy [47]. 

Samples for SCRS need to be prepared and processed accordingly to avoid spectral interference and maintain bacterial cells intact. Therefore, crude homogenates require additional concentration and isolation procedures. In order to correctly classify SCRS, a corresponding dataset and correct model are required. The right choice of bacterial cells and their preparation is crucial in establishing a correct dataset [48]. 

### 5.3. Mass Spectrometry Methods 

Mass spectrometry (MS) is a group of techniques based on the ionization of (bio)molecules, separation of ionized products in a vacuum environment, detection and quantitation of their mass-to-charge (*m*/*z*) ratio and subsequent generation and analysis of mass spectra [49]. Initially applied to small molecule chemicals, in recent years, the focus of MS applications has shifted to biomacromolecules (proteins, nucleic acids, etc.), which require mild molecular ionization methods, such as electrospray ionization (ESI) and matrix-assisted laser desorption ionization coupled with time-of-flight detection (MALDI-TOF) mass spectrometry [50]. MALDI-TOF MS is based on the simultaneous desorption and soft ionization of a sample-matrix mixture in which unfragmented peptides are detected. The matrix, which co-crystallizes with the sample, absorbs energy from short laser pulses and releases ions of intact peptides from the sample [51]. This creates a spectral fingerprint in which whole cell proteomes are analyzed, typically within an *m*/*z* range of 2–200 kDa [51]. Similar to Raman spectroscopy, MALDI-TOF MS relies on pre-existing spectral libraries in order to correctly analyze a spectral fingerprint. Current MS libraries are clinically oriented, and only a few of them are available for food safety applications [50]. 

Thus, it was proposed to combine MALDI-TOF MS and bioinformatics to create custom libraries for the analysis of raw milk isolates. When compared to 16 S rRNA amplicon sequencing, similar clustering patterns and significant discriminatory power were observed [52]. A combination of MALDI-TOF MS proteomics and gas chromatography–mass spectroscopy (GC-MS) metabolomics was able to identify three red meat pathogens directly from enrichment broth, thus allowing for further development of this method as a diagnostic tool [51]. MALDI-TOF MS and 16 S rRNA amplicon sequencing were used together to characterize the microbial populations of wild boar meat [53]. 

The main limitations of MS are the need for expensive and bulky equipment and extensive sample preparation. Furthermore, MS fingerprints must be compared against a pre-existing database; therefore, new species cannot be identified. 

### 5.4. Calorimetry 

Calorimetry measures heat release into the environment by a chemical or biological sample. Bacterial growth and metabolism can also be monitored via microcalorimetry [54], and there is no limitation on the sample matrix as long as heat can be transferred [55]. The sample is placed into a closed ampoule within a controlled isothermal chamber at a fixed temperature where the heat exchange between the sample and the surrounding environment is measured [56].

Isothermal microcalorimetry (IMC) was investigated for the factors that determine the detection time of aerobic microbial contaminations, namely: (i) an initial number of bacteria; (ii) performance of the microcalorimeter and (iii) the provision of oxygen in solid or liquid media. It was found that an increased number of bacteria via membrane filtration reduced the detection time as well as setting correct threshold limits. Additionally, solid media provided a greater availability of oxygen for growing bacteria than liquid due to the slow diffusion of oxygen from headspace to the cells [54]. 

IMC was also used to determine the minimal inhibitory concentrations (MICs) of antibiotics in raw mixed cultures derived from Swiss hard cheeses grown in milk. Changes in metabolism due to the efficacy of antibiotics produced distinct heat flow curves which allowed for the determination of MICs. IMC allowed for the direct measurement of samples without additional preparation; however, an extensive calibration of 2 days was required [55].

## 6. Oxygen Sensor-Based Respirometry in Microbial Testing

### 6.1. Measurement Principles 

Optical O_2_ respirometry is a group of techniques that uses phosphorescent O_2_ sensing materials in the form of solid-state coatings or soluble probes to trace the dynamics of O_2_ concentration in biological samples, usually containing living cells [57]. The long-decay emission of these materials is reversibly quenched by O_2_ via a collisional (i.e., non-chemical) mechanism, and this process reduces sensor intensity and lifetime signals in a manner dependent on O_2_ concentration [57,58,59]. The relationship between the sensor signals and O_2_ concentration is described by the Stern–Volmer equation [60]:

*I_o_*/*I* = *τ_o_*/*τ* = 1 + *k_q_* × *τ_o_* × [*O*_2_] = 1 + *K_SV_* × [*O*_2_] (1)

where *I_o_*, *I* and *τ_o_*, *τ* are the phosphorescence intensity and lifetime, LT, signals in the absence and presence of *O*_2_, respectively; *k_q_* is the bimolecular quenching rate constant and *K_SV_* is the Stern–Volmer quenching constant. Thus, *O*_2_ concentration or partial pressure can be quantified by measuring sensor *I* or *τ* signal [60]:

[*O*_2_] = (*τ_o_* − *τ*)/(*τ* × *K_SV_*) = (*I_o_* − *I*)/(*I* × *K_SV_*) (2)

**Figure 1:**
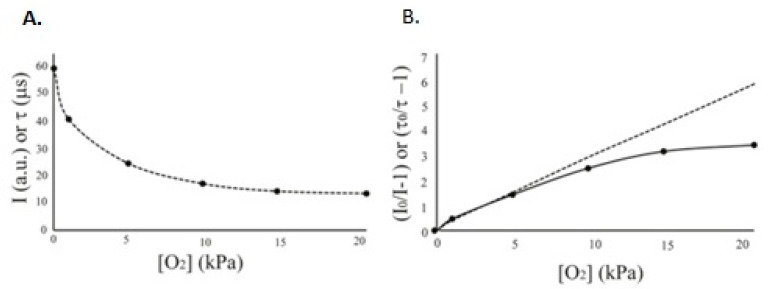
Typical relationship between sensor *I* and τ signals and O_2_ concentration (**A**) and its linearization in Stern–Volmer plots (**B**) which show the theoretical straight line (dashed) and experimental curve with data points (solid line). Reproduced from [62] with permission of Elsevier.

The dependence between O_2_ concentration and sensor parameters is shown in Figure 1. The linear plot corresponds to an ideal system with homogenous dispersion of dye molecules within the sensor matrix [60]. However, in practice, curved plots reflecting heterogeneous dispersions are commonly observed, which are better described by the ‘two-site’ model [58,61].

Phosphorescence intensity measurements are used in some O_2_ sensing platforms, but they are more error-prone, unstable and difficult to operate. This is because intensity signals, I, are influenced by the fluctuation of the light source and detector, sensor positioning/measurement geometry, photobleaching and leaching of the dye, optical properties of the sample and instrument variability [57,58,59,60,61]. This in turn leads to large measurement errors, unstable calibrations and inaccurate results.

In contrast, LT (or τ) is the intrinsic parameter of the sensor material [60], which is influenced by O_2_ concentration (see Equations (1) and (2)) but is independent of the instrument, measurement settings and dye concentration [58]. These features of LT-based O_2_ sensing allow for more stable and robust measurements, including respirometry [57,63], with batch-calibrated disposable sensors and sensing systems. 

LT-based O_2_ sensing can be performed in the time domain whereby short excitation pulses are applied on the sensor/sample, and its emission decay is traced directly [60]. A simplified method called rapid lifetime determination (RLD) [64] uses time-resolved fluorescence intensity (TR-F) measurements at two different delay times with subsequent calculation of LT values using the following equation [57,63]:

*τ* = (*t*_2_ − *t*_1_)/ln(*I*_1_/*I*_2_) (3)

where *t*_1_, *t*_2_ are the first and second delay times, and *I*_1_ and *I*_2_ are the corresponding TR-F intensity signals. The TR-F and RLD modes are supported by standard multilabel readers, although their temporal resolution (LT values > 15 μs) is compatible with only some sensor materials. 

In phase-domain methods, the sensor is excited with periodically modulated light while measuring the phase shift, Δ*ϕ* (degrees angle), of the emission signal [65,66]. Phase readout is a version of LT-based sensing, as Δ*ϕ* is related to *τ* as [60]:

tan(**Δ*ϕ***) = 2 × *π* × *ν* × *τ*, or (4)

**Δ*ϕ*** = atan(2 × *π* × *ν* × *τ*) (5)

where *ν* is the modulation frequency of excitation (Hz). 

### 6.2. O_2_ Sensing Materials 

While many different photoluminescent dyes have been suggested for O_2_ sensing [57,59], the dyes currently used in biological applications and O_2_ respirometry are phosphorescent Pt(II)-porphyrins (excitation and emission bands are 400/650 nm or 525/650 nm) and Pt(II)-benzoporphyrins (430/760 or 615/760 nm); Ir(III)-porphyrins (390/655 or 525/655 nm) [67] and fluorescent complexes of Ru(II) (470/615 nm) [57,58]. These dyes and materials on their basis have appropriate O_2_ sensing characteristics, brightness, chemical and photo-stability, commercial availability and price. However, they differ in their spectral and LT characteristics, compatibility with the common sensor matrices (polymers, other materials) and available detection instrumentation. When such an O_2_-sensitive dye is embedded in a suitable matrix or microenvironment, it produces sensing material with a characteristic and stable response to O_2_. 

The main types of sensor materials are (i) solid-state, i.e., water-insoluble, sensors, and (ii) liquid and water-soluble probes. The solid-state sensors are integrated into respirometric systems either as permanent coatings on the inner side of assay vessels [59] or as small inserts with dot sensor coatings immersed in food packs, homogenates or liquid samples [13,68]. The coatings usually comprise a hydrophobic dye dissolved in a hydrophobic polymer; they are produced from a precursor ‘cocktail’ with sensor ingredients, dissolving them in organic solvent and spotting on a suitable substrate or assay vessel [57]. Alternatively, monomeric precursors can be polymerized or cured (e.g., sol-gels, poly(acrylamide), styrene). Multistep, multicomponent fabrication and coating procedures for solid-state sensors are far from trivial, especially when uniform and stable; batch-calibrated and disposable sensors or coated substrates are required by large-scale applications. 

Soluble O_2_ probes comprise dispensable liquid reagents, which are simply pipetted to aqueous samples in an assay vessel. Such probes are represented by [57]: (i) small molecule structures, i.e., hydrophilic derivatives of the above phosphors; (ii) macromolecular conjugates, e.g., a reactive derivative of the dyes (e.g., PtCP-NCS) covalently linked to a hydrophilic carrier, such as serum albumin and (iii) stable dispersions of micro- and nanoparticles, such as core–shell nanoparticles of amphiphilic polymers impregnated with hydrophobic dye molecules. 

The soluble O_2_ probes overcome the challenges of solid-state sensors associated with their fabrication and integration in respirometric substrates and assays. They are better suited for use in high throughput cell-based screening assays of small samples on standard bioassay substrates and equipment (i.e., microrespirometry). However, liquid probes have their own challenges, such as (i) being poorly usable in large samples (costly); (ii) being more susceptible to optical and quenching interferences than ‘shielded’ solid-state sensors and nanoparticles [69]; (iii) unwanted interactions with cells and surfaces during the assay (e.g., non-specific binding); (iv) long-term storage in liquid form or drying/reconstitution issues and (v) complex synthesis. 

The selection criteria of the different sensor materials and their uses in respirometric systems and assays will be elaborated in more detail in the sections below. 

### 6.3. Typical Profiles in Optical O_2_ Respirometry and Their Analysis 

**Figure 2:**
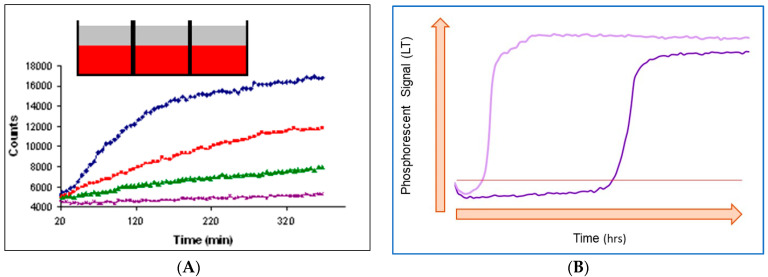
Typical respiration profiles produced by mammalian cells (**A**) and bacterial cells (**B**). Cell numbers are increasing from bottom to top in (**A**) and from right to left in (**B**). The inset in (**A**) shows that samples in microwells are sealed with mineral oil (top layer). The horizontal line in (**B**) is the signal threshold used to identify threshold time (TT) values (see below).

Depending on the sample, assay format and measurement settings, O_2_ sensor-based respirometry can produce respiration profiles of different shapes [57,63]. The two main types, characteristic of mammalian cells and bacterial cells, are shown in Figure 2. It is worth noting though that technically (i.e., by changing assay settings: cell density, timing, etc.), it is possible to convert one type of profile into another. Some ‘special’ formats are also known, such as the monitoring of adherent mammalian cells pre-stained with an intracellular O_2_ probe in an open microplate under a steady state [70], which we will not discuss here.

#### 6.3.1. Mammalian Cell Respiration 

Respiration profiles of mammalian cells (and this is true for many other assays with enzymes, microtissue samples or small organisms [71]) usually have a relatively short time span (5 min–2 h) close to the linear initial part and curved final part with saturation (Figure 2A) [57,72]. This shape reflects that the biomass is not changing much during the assay (cell doubling times 24 h) and that the OCR is low and constant. 

However, small and weakly respiring samples exposed to ambient air cannot develop O_2_ gradients due to the high rate of atmospheric O_2_ back-diffusion [57]. Therefore, for the measurements in standard 96/384-well plates, mineral oil is applied on top of each sample (see inset in Figure 2A). Such a seal, applied with a pipette or a simple dropper, creates a barrier for ambient O_2_ diffusion and also primes the assay [72]. The same general approach is used in sealable plates [73], microchamber devices [74] or fluidic biochips [75] specially designed for respirometry. Besides the hermetic sealing, these devices also provide a high cell volume-to-sample volume ratio, which facilitates fast, sensitive and accurate OCR measurements [73]. 

Usually, the linear part of the respiration profile is used for OCR calculation, for which:

OCR = Δ[*O*_2_]/Δ*t* (6)

OCR(*t*) = Const = *v_o_* × *N*/*V* (7)

where *v_o_* is the specific respiration activity of a cell, *N* is the number of cells being measured and *V* is the measurement volume. 

Profiles expressed in the [O_2_] concentration scale potentially allow for the determination of absolute OCR values (e.g., in pmol/min/cell). Changes in OCR at different time points or O_2_ levels can also be assessed from full profiles. However, the sealing efficiency of the measurement chamber, the rate of O_2_ back-diffusion and the sensor calibration equation (described above) must be considered. All this leads to complex mathematical models and data processing algorithms for such sensor systems [76]. 

Another common approach in respirometric assays is to deal with raw sensor signal (*τ* or even I) profiles and corresponding relative OCRs and their changes [72]. This approach simplifies data processing by avoiding complex modeling and errors brought in by signal conversion and profile transformation. 

The temperature has strong effects on cell respiration, sensor signals and response to O_2_ (stronger quenching at high temperatures [60]). So, if samples were prepared on a bench at room temperature and then set for measurements at 37 °C, gradual temperature equilibration of the sample will take place, seen as a downward drift of the sensor signal and skewed respiration profile in the first 15–20 min [77,78]. If significant, this part of the profile should either be excluded from analysis or eliminated by preparing the plate with samples on a heated stage or pre-incubating at 37 °C prior to the sealing and measurement. Table 1 summarizes the typical assay settings used in mammalian cell respirometry and compares them with bacterial cell respirometry.

#### 6.3.2. Respiration Profiles of Bacterial Cells and their Characteristic Features 

Typical respiration profiles of aerobic bacterial cells shown in Figure 2B differ considerably from those produced by mammalian cells, as do their assay settings (Table 1). These profiles usually span over several hours and have a sigmoidal shape with a steep transition of the sensor signal from low values to high [79]. Such a shape reflects the steady increase of the biomass (exponential with cell doubling times of 20 min) and respiration rate during the assay. 

In the initial phase (flat part on the left side), cell numbers and OCRs are low and cannot change the sample oxygenation state which remains air-saturated. Phase 2 kicks in when the sample reaches a certain cell density, which prompts fast depletion of dissolved O_2_. This is seen as a steep increase in the sensor signal (I or *τ*) from the low baseline level to the high level. Once the dissolved O_2_ is depleted, the signal enters Phase 3, which is also flat but much higher and corresponds to a deoxygenated sample. 

The steep transition from the air-saturated state to deoxygenated state upon achieving high CFU and OCR values makes bacterial respirometry more tolerant to back diffusion of atmospheric O_2_. For small samples measured in standard microplates, mineral oil sealing is still required, as it makes sensor signals more stable and respiration profiles smoother and less affected by mechanical movements of the plate during reads. Whereas for larger samples measured in plastic tubes, special sealing becomes unnecessary [79,80]. The liquid sample itself provides a sufficient barrier for O_2_ diffusion from the air phase on top of the tube to the bottom part where the sensor is usually placed. Still, rigorous handling, agitation or shaking of samples, which affect their oxygenation and sensor signal, should be avoided during the assay. 

The characteristic onset of the sensor signal for samples with a higher initial load of bacteria occurs faster than for samples with a lower cell load (Figure 2B) [78]. This is because during Phase 1 and the start of Phase 2, the number of cells in the sample, N, and the OCR continues to increase exponentially over time, while the stock of O_2_ is limited:

N(t) = *N_o_* × 2^*τ*/*t_d_* (8)

OCR(t) = (*v_o_* × *N_o_* × 2^*τ*/*t_d_*)/*V*) (9)

where *N_o_* is the initial (seeding) number of cells, *t_d_* is the cell doubling time and t is the current time since the start of the assay. 

Of note, low O_2_ levels in the sample in Phases 2/3 may trigger other processes, such as (i) inhibition of respiration, i.e., a reduction of *v_o_*; (ii) anoxic cell death reducing the number of respiring cells, *N*, and (iii) increased O_2_ back-diffusion due to a higher gradient. These factors can alter the classical sigmoidal shape and produce more complex respiration profiles [69]. However, the initial part of Phase 2 of the profile for which dissolved O_2_ levels still remain close to ambient O_2_ is usually stable and less affected by such factors. Therefore, it is better suited for analysis (as will be shown below). 

Being a rather complex and floating function (Equation (9)), OCRs of microbial samples are less commonly used as assay readouts, whereas the initial load of viable bacteria, *N_o_*, which is equivalent to total aerobic viable counts, TVC, is a more useful parameter for many applications. *N_o_* and TVC are amenable to simple quantification by O_2_ respirometry [78,81] since they determine the onset time on the respiration profile or the time to reach a certain threshold signal (*TT*) [82]. Such a threshold signal is shown as a red line in Figure 2B. 

Furthermore, it appears that in most TVC assays, sample *TT* is linearly related to its log(TVC) value [78]:

*TT* (h) = a − b × (log (CFU/mL)) (10)

**Figure 3:**
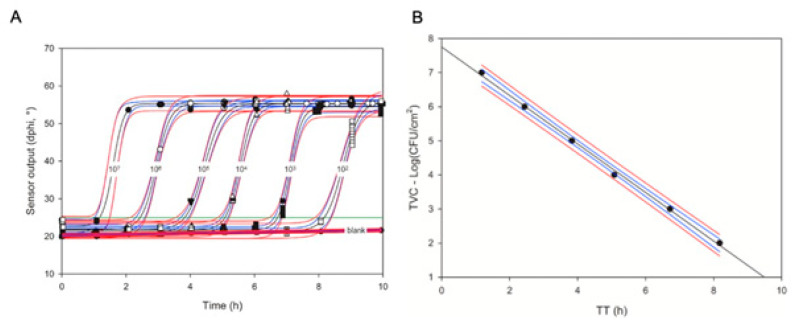
Respiration profiles of *E. coli* standards in nutrient broth (NB) media generated in swab sensor vials in 4 replicates (**A**) and the resulting calibration plot for the TVC assays of swabs (**B**). Note: This figure is reproduced from [80].

A mathematical equation for such a relationship can be established in a simple, once-off calibration experiment with a set of TVC standards (see Figure 3 and [81]). The semi-logarithmic linear relationship between *TT* and TVC (Equation (10)) is convenient and works well with various microorganisms and sample types, including crude homogenates of solid food products [68,81,82,83]). It spans over many Logs of cell concentrations and does not require additional dilutions and re-calibrations. Individual signal readings (τ or Δϕ) and corresponding respiration profiles are stable, reproducible and uniform so that individual sensor readings can be taken at a low frequency (every 20–30 min) and fitted with sigmoidal functions to produce smooth profiles and accurate *TT* values [79]. 

The general procedure of a typical respirometric TVC assay looks as follows:

- Take sample specimen and put it in assay medium that promotes microbial cell growth; homogenize, if required;
- Transfer the sample to a measurement chamber and record its sensor signals over time under standard (optimal) assay conditions;
- Determine (once-off) the optimal threshold sensor signal for these conditions and the recorded respiration profile(s), which accounts for sensor, sample and assay variability and avoids any false-positive or false-negative results;
- Apply the threshold and determine the corresponding *TT* value for each profile and sample;
- Apply the mathematical equation (pre-determined, see above) to convert measured *TT* values into corresponding cell counts (log (CFU/mL) or log (CFU/g) for each tested sample.

In theory and experimentally, microbial respirometry can detect a single viable cell in a sample [78]. However, practical sensitivity and LOD (CFU/mL) are limited by several factors: (i) sampling errors due to statistical variation at low cell numbers, (ii) measurement errors due to variability of respiration profiles and *TT* values (in repeating experiments); (iii) sample volume and (iv) sample dilutions used. Therefore, larger (and so more representative) samples together with a low dilution factor provide a higher practical sensitivity and lower LOD. This is illustrated in Table 2. 

In contrast, O_2_ respirometry of individual mammalian cells is very challenging due to their very low OCRs. Although some studies achieved this in sophisticated biochips [74]), standard assays usually operate with high cell numbers > 1000, typically 20–100 k cells per well (see Table 1).

Due to their high sensitivity, respirometric TVC assays based on the determination of *TT* values are gaining wide practical use, particularly in low TVC and sterility testing, industrial hygiene, process control, food safety and environmental monitoring [57,80]. These simple mix-and-measure assays are rapid, cost-efficient and applicable to different types of samples and for many important microbiological and analytical tasks. Their time-to-result is reciprocal to the microbial load: 1–2 h for bacterial loads 10^8^–10^6^ CFU/mL or 8–10 h for very low TVCs [79]. 

Respirometric TVC assays can also provide a simple semi-quantitative assessment of samples with a single end-point read of their sensor signals. In this case, the measurement time point from the start of the assay is calculated from the existing calibration for the given TVC threshold (CFU/mL) and sensor signal threshold (μs, or Δϕ). This method allows the operator to differentiate measured samples into three categories: those with sensor readings below the threshold are graded as negative or ‘clean’; those with readings above the threshold are graded as positive or ‘contaminated’. In addition, if the sensor reading falls close to the threshold, the sample is graded as suspicious and requires further assessment, e.g., another sensor reading. Overall, this gives a useful ‘traffic light system’ for industrial samples (e.g., food, dairy products) with fast testing and a Go/No-Go decision on batch release on the same day or even shift [82]. 

Besides the initial load of aerobic viable cells (N_o_, TVC or CFU/mL), other parameters also influence the shape of the sample respiration profile and *TT* value (Equation (10)). These parameters include (i) the type of cells, their v_o_ and t_d_ characteristics and metabolic status; (ii) assay conditions—media, temperature, mass exchange and (iii) other compounds or treatments applied [84,85]. These parameters can also be assessed by O_2_ respirometry. 

**Figure 4:**
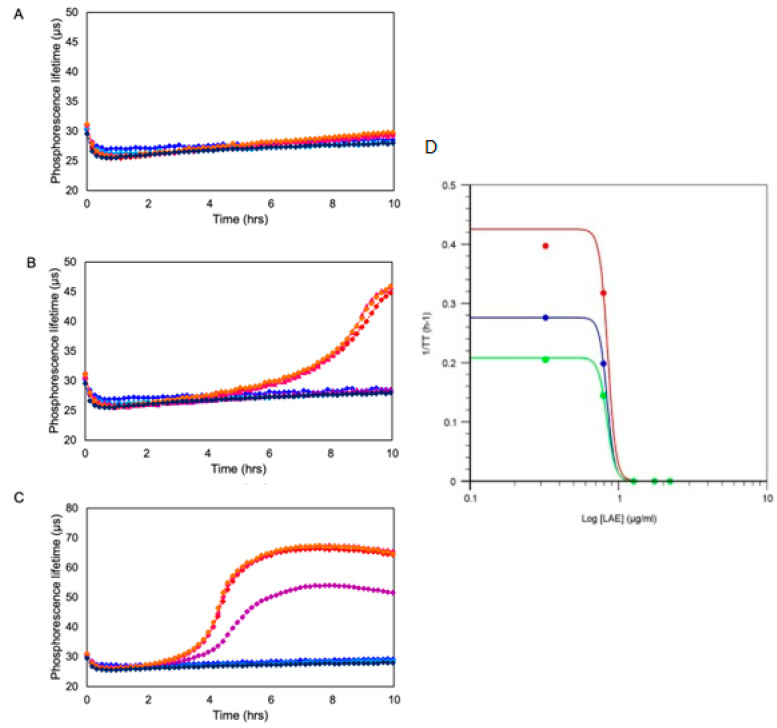
Exemplary profiles of toxicity of an antimicrobial compound (LAE) produced by O_2_ respirometry of Gram-negative bacteria *Pseudomonas fluorescens* taken at initial concentrations of 10^4^ CFU/mL (**A**), 10^5^ CFU/mL (**B**) and 10^6^ CFU/mL (**C**) in the presence of increasing concentrations of LAE (2.1, 6.3, 18.9, 56.7 and 166.7 µg/mL). Profiles were generated on Platform 4 using MitoXpress probe. This figure is reproduced from [85].

One such application widely used by the industry and research labs is the toxicological screening and profiling of compounds, such as antimicrobials, food additives, drugs and drug formulations and complex environmental samples [71,84,85]. In such applications, microbial and mammalian cell O_2_ respirometry provides fast, quantitative and accurate assessment and determination of compound EC50 or MIC values under various conditions. This in turn gives researchers and food and pharma companies rich information on the mode and mechanism of the toxic action of compounds on cells, including cell specificity, dose and time dependence of toxicity, effects of mixtures and drug formulations (additive synergistic or antagonistic), in a very fast, quantitative and accurate manner. Examples of such data are given in Figure 4. 

Sensor material type, sensitivity to O_2_ and calibration function also have an effect on respiration profiles, mostly on the magnitude of signal change. The shape of the profile is less affected if the O_2_ calibration covers the range 0–21 kPa or 0–200 μM of O_2_ (Figure 1). 

On the other hand, sensor materials can show chemical and photo-toxicity on cells [86] and thus influence the respirometric assays and measured *TT* and OCR values. Conversely, certain chemical ingredients present in complex and even regular growth media may affect the sensor/probe signals [87]. Thus, several pH indicators, chromogenic substrates, metal ions, surfactants and aromatics present in common selective media were shown to cause optical and quenching interferences [69]. Such effects, if seen, should be tackled by optimizing the sensor material and assay settings. In this regard, ‘shielded’ solid-state sensors look more favorable than soluble probes [69]. 

Generally, the high analytical performance of the sensors, including stable O_2_ sensing characteristics, calibration-free operation, no cross-sensitivity, stability to optical and chemical interferences and sample matrix effects and general ruggedness, is key for most applications of respirometry, particularly the measurement of absolute OCRs, TVC testing and safety assessment of food products, process hygiene, antimicrobials and high throughput screenings of new chemical entities, drugs and formulations. Disposable respirometric devices for such applications, including sensor tubes [79], biosensor sachets and swab vials [80], which provide calibration-free operation, are becoming popular and abundant. These off-the-shelf devices are usually batch or factory calibrated, quality-controlled and optimized for specific applications and produced in batches of >100 s by the industry and in research labs [79]. All these features of O_2_ sensor-based bacterial respirometry allow it a broad range of applications, many of which are important and unique. 

### 6.4. Existing Formats and Applications of Bacterial Respirometry

A brief summary of the main O_2_ sensor-based detection platforms dedicated to or usable in bacterial cell respirometry and related applications is given in Table 3.

Platform 1 was designed by Becton Dickinson for the detection of microbial infections (*Mycobacterium tuberculosis*) in blood samples [88]. Samples were dispensed into sterile measurement vials with liquid growth media and sensor coatings (ruthenium dye in silicone resin) at the bottom, then capped, placed in an incubator rack and monitored for their intensity signals with a fluorescent detector. The system was simple but slow (detection took several days), bulky, specialized for one application and performed qualitative or semi-quantification analysis (growth/no growth, fast/slow). Its low sensitivity and speed were due to the slow-growing test organisms and also air in the vial headspace. A similar system with a fluorescent CO_2_ sensor was observed to function more optimally [88]). 

Adaptation of Platform 1 for operation in 96/384-well plates gave rise to Platform 2, called BD Biosensor [89], which was more flexible and user-friendly and tailored for widely available fluorescent readers. Still, the main settings of Platform 2 were not optimal for quantitative O_2_ sensing and respirometry. Its main shortcomings were the silicone - Ru-dye sensor chemistry, intensity-based sensing, no sample sealing, slow response, low sensitivity and biocompatibility unsuitable for adherent cells. Nevertheless, this platform was successfully demonstrated in the screening of bacterial cells and antimicrobials but was later withdrawn from the market. 

The Platform 3 OxyDish Sensor Plate [90] is an improved version of the BD Biosensor with Pt-porphyrin-based sensor coatings in 24/96-well plates. The sensors have better performance and biocompatibility, and the plate operates with a portable multichannel phase detector, which provides stable operation with a quantitative real-time readout of O_2_ concentration and simultaneous measurement of multiple samples. 

Platform 4 was designed for sensitive multiparametric analysis of adherent mammalian cells using special sealable microwell plates and O_2_ and pH sensors on the tips of the pins that go inside the wells and mechanically seal the samples [76]. While showing excellent performance and usability in various metabolic and bioenergetic studies performed with mammalian cells [73], Platform 4 is poorly suited for bacterial cell respirometry, as it is overly complicated, inflexible and expensive for basic TVC assays and food applications.

Platform 5, also marketed as MitoXpress^®^ and GreenLight-960 systems [72,81,82], comprises a dedicated but flexible DIY (Do-It-Yourself) approach to O_2_ respirometry [85]. It differs from Platforms 1–4 in that it uses:

(i) A soluble, dispensable O_2_-sensing probe MitoXpress-Xtra (Agilent) based on a Pt-porphyrin dye instead of the pre-made, solid-state sensor coatings;
(ii) Standard 96/384-well plates, uncoated, tissue-culture treated and even customized assay substrates [74];
(iii) Sealing the samples on the plate with mineral oil;
(iv) Measuring the plate on a multilabel plater reader in the TRF/RLD mode [77], thus implementing LT-based O_2_ sensing.

In the assay, the medium, samples with cells and the probe are simply dispensed into the wells, covered with mineral oil, placed in a TR-F reader and measured at constant temperature (30 or 37 °C) periodically to generate respiration profiles for each sample. Platform 5 has been demonstrated in many useful applications, including mammalian cell respiration and responses to stimuli [77], enumeration of bacteria in pure cultures [78], TVC in complex food and environmental samples [81], screening of antimicrobials and drugs with EC50 and MIC determination [85] and metabolic and toxicological profiling of bacteria and environmental samples [71,84].

Platforms 6–9 rely on disposable plastic substrates (vials, sachets, swab vials) integrated with solid-state O_2_ sensitive coatings or inserts based on bright near-infrared dye PtBP [79,80]. The sensors in assay vessels are interrogated with a portable and low-cost LT-based detector. Platforms 6–9 were developed for TVC testing of complex samples, such as raw meat, mince and other foods (solid and liquid) and their crude homogenates and swabs. They differ in their:

- Measurement vessels type and size: 2 mL vials for Platform 6, 30–50 mL vials for Platform 7, 15 mL vials with swab brushes for Platform 8 and flexible plastic pouches for Platform 9;
- Sensor integration method: permanent coatings (dots at vial bottom) for Platforms 6, 7 or on vial side for Platform 8 or small inserts membrane type in Platform 9;
- Detector type: automated benchtop reader with incubator and carousel for sensor vials (GreenLight-930 in Platform 6) or autonomous handheld reader for in-field operation (Platforms 7–9).

Platform 10—the *ambr* system from Sartorius [92,95]—uses essentially the same sensor chemistry and settings as Platform 3 (OxyPlate). It was designed for rapid development, optimization and up-scaling of industrial biofermentation processes, particularly the high throughput optimization of media composition, conditions of cell growth and product harvesting in small-scale model bioreactors. It is dedicated to achieving maximal rates of cell growth by working at maximal cell densities and expression levels of the target product. Similar to Platform 4, Platform 10 serves rather special niche applications; therefore, it is difficult to characterize it with respect to bacterial cell respirometry and TVC assays. The same is true for the remaining Platforms 11 and 12. 

Automated Platforms 1, 2, 4, 6 and 10 provide high throughput (e.g., up to 48 samples per run for Platform 6), while manual Platforms 7–9 can handle up to 20–30 samples per run, which is quite sufficient for on-site TVC testing. TVC assay throughput is limited by sample preparation time, which, for the assays with synchronous measurement of all samples, should be kept below 15 min [79]. However, sensor measurement takes only 1–3 s per sample or <1 min for the whole batch, so one reader can be used to measure several batches with shifted assay start time. 

Additional equipment includes just media and a pipette/dispenser for Platform 6, a simple incubator or block heater for Platforms 7–9 and a heat-sealing machine for Platform 9. Detection equipment for O_2_ respirometry is mostly LT based, which provides stable and accurate measurement of sensor signals and resilience to sample matrix effects and optical interferences that may occur in TVC assays [69]. These features are also aided by the use of indicator dyes with high brightness and longwave spectral characteristics, such as PtBP used in Platforms 3, 6–9 and 11. 

### 6.5. Comparison of O_2_ Respirometry with Established Microbial Testing Methods

From the previous sections, it is clear that O_2_ sensor-based respirometry techniques have enabled a range of new bioanalytical applications. The most important and demanding microbiological applications include (i) rapid TVC testing of complex samples, such as food, environmental, clinical samples and swabs; (ii) compound screening and toxicological profiling of antimicrobials, drugs and drug formulations, libraries of mutant cells and culturomics and (iii) sterility and hygiene testing via surface swabs (Table 3). In addition, with respect to microbial and TVC testing, O_2_ sensor-based respirometry possesses a number of useful features and advantages over the alternative methods currently in use (Table 4). 

The main competitive advantages and operational characteristics of the respirometric TVC assays include:

- Detection and enumeration of only *viable* cells via monitoring of their growth.
- Ultimate single cell sensitivity, broad concentrations range and minimal number of steps.
- Rapid mix-and-measure assay in liquid media with real-time signal output and time-to-result 1–10 h. Quantitative, accurate and automated.
- Assays require only disposable sensor vials, a simple sensor reader and an incubator. No special facilities, equipment or skills are needed.
- Various types of samples can be analyzed: swabs, crude homogenates, food and environmental samples, etc. Sample preparation is the same as in the ISO method.
- Choice of different sensor materials, assay substrates (microplates, vials, pouches) and detectors.
- Portable, autonomous, low-cost commercial sensor readers that provide LT-based sensing and low start-up costs for the whole system (from $2000 upwards).

The ‘gold standard’ agar plating TVC methods (ISO 4833-1:2013 and ISO 18593:2018 [6,7]) and respirometry both detect *live aerobic* bacteria, but the ISO method uses the growth of colonies on *solid* agar media in multiple plates (serial dilutions) and tedious end-point counting. As a result, the ISO test is much slower than respirometry (24–72 h vs. 2–8 h) and is more complex and laborious (dilutions, multiple steps). While applicable to complex samples without major pre-treatment (only homogenization), the ISO test is poorly automated and produces lots of hazardous waste. Both tests allow for the predictive identification of particular bacterial specie using special *selective* media [69], but still, these results require confirmation by molecular methods. 

The ATP-BL assay is sensitive (down to 10 cells/mL), simple and fast. The detection step uses one reagent, which provides cell lysis and measurement of the BL signal on a simple instrument. The assay is not selective; it detects mostly viable bacterial cells, but somatic cells and lethally injured bacteria can also contribute to measured ATP signals. Moreover, the relationship between ATP (or BL signal) and TVC is not simple and depends on the sample and cell type. BL detection suffers from matrix effects and requires clear samples (diluted or processed). Therefore, lengthy steps of sample clarification and enrichment are often used [15,18].

DNA/RNA amplification assays have long and tedious sample preparation steps that include enrichment of target cells, cell lysis and RNA extraction and purification followed by RT-PCR. While providing the best selectivity and identification of specific strains of bacteria, such assays cannot reliably distinguish dead and live cells or provide single-cell sensitivity. They are expensive, prone to matrix effects and false-negative results and require special facilities and equipment. More fast or automated methods, such as qPCR and LAMP, can simplify the assay but only in part. The 16 S rRNA sequencing reports on the whole spectrum of bacterial species present in the sample, but it also includes tedious sample preparation steps, shares similar limitations with the PCR and has moderate sensitivity. O_2_ respirometry does not have all these limitations, but it does not provide the selectivity of the molecular methods.

The antibody-based TVC assays, such as live cell ELISAs, lateral flow and IMS assays, show selectivity similar to PCR, but lower sensitivity and more prominent matrix effects. Sample preparation and throughput are similar to respirometry, while detection equipment is even simpler (colorimetric, visual or with digital cameras). But again, these methods cannot distinguish between dead and viable cells. 

The last group of instrumental techniques—flow cytometry, Raman/SCRS and MS—provide high selectivity (species identification potential) and sensitivity (rarely reaching 1 CFU/mL levels). However, they all require expensive and stationary detection equipment, the corresponding infrastructure (dedicated labs) and trained personnel. The assays also require special sample preparation procedures and data analysis algorithms.

## 7. Conclusions

The above sections outline the current methods available for the detection of microbes and pathogens in the food industry and related areas. The established methods tend to rely on laborious procedures and sophisticated laboratory equipment, have lengthy times to final result and require skilled personnel in a centralized or external lab away from food processing facilities. Although some automated and/or portable devices are available (such as the ATP kit and Tempo and Soleris systems), there is a strong demand from the food industry and research and diagnostic labs for new analytical systems and rapid detection and quantification of microbial pathogens in food and environmental samples. The O_2_ sensor-based microbial respirometry is well positioned here, as it provides a number of simple and versatile analytical solutions and measurement platforms, particularly for rapid TVC testing of complex food samples, crude homogenates, environmental samples and swabs, sterility testing, screening of antimicrobials and selective media optimization (culturomics). These sensor systems also have competitive advantages over the traditional microbial testing methods in terms of the time-to-result (2–8 h vs. 24–48 h for the ISO method), simple mix-and-measure assay procedures, no special skills, portable low-cost sensor readers and sensor chemistry, robustness and suitability for in-field deployment and use.

A number of sensor-based TVC testing systems are already in practical use by the industry and food safety and research labs, and their use and diversification continue to expand rapidly. They operate with convenient, disposable and low-cost sensor vials, swab vials and sensor sachets, which provide more simple, fast, versatile and cost-efficient alternatives to the established conventional testing methods. Therefore, respirometric microbial cell assays are performed with different types of samples and in many areas, including basic microbiology, food science and environmental monitoring.

Future directions for O_2_ sensor-based microbial respirometry include its extended demonstration and validation with various types of food products; development of selective tests for the key food-borne pathogens (e.g., *Lysteria*, *Salmonella*, *E. coli* O157:H7) using dedicated selective media; further diversification customization and automation of existing systems for food research and microbial safety assessment.

## Figures and Tables

**Table 1:** Comparison of the typical settings for the two main types of O_2_ respirometry.

Parameter	Mammalian Cell Respirometry (1)	Bacterial Cell Respirometry (2)	Comments
**O_2_ Sensor/probe type**	Various, soluble or solid-state	Various, soluble or solid-state	Interchangeable, different dyes
**Assay substrate**	Microwell plates, special substrates	Standard plates and plastic tubes, sachets	More critical for (1) than (2)
**Sample sealing options**	Oil seal, sealable microchambers	Oil seal or no seal (liquid barrier)	Critical for (1) Less critical for (2)
**Assay volume and sample type**	0.05–1 mL, simple and uniform	0.1–50 mL, complex and variable	Larger for (2)
**Cell number range**	10^4^–2 × 10^5^ per well	Single-cell—10^7^ cells/mL	May vary for the different apps
**Assay T, °C**	37 °C	7 °C, 30 °C, 37 °C	Cell-specific
**Instrumentation**	Benchtop reader with T-control	Handheld reader + Incubator	Stationary vs transportable
**Measurement time**	5 min–2 h	1–16 h	Longer for (2)
**Frequency of reads**	1–5 min Or end-point reads	10–60 min Or end-point reads	Slower for manual readers
**Repeated measurements**	Desirable and common	No	Samples discarded
**Signal monitored**	Int, LT, [O_2_]	Int, LT, [O_2_]	LT is preferred
**Profile shape**	Linear or extended sigmoid	Steep sigmoid	May be different in some apps
**What parts are analyzed and how**	Linear part–signal slope or [O_2_] slope	Signal onset—time to threshold, TT	
**Calibration used**	Sensor LT vs [O_2_]	TT vs log(CFU/mL)	Once-off calibrations
**Assay readout**	Absolute or relative OCR values	Cell counts—CFU/g or CFU/mL	May vary in different apps

**Table 2:** Estimation of LODs for the different samples and assay conditions.

Assay Volume	LOD_1_ for 1 CFU/Sample	LOD_10_ for 10 CFU/Sample	LOD_10_ plus 1:10 Sample Dilution	Application
**0.1 mL**	10 CFU/mL	100 CFU/mL	1000 CFU/ml	Screening
**1.0 mL**	1 CFU/mL	10 CFU/mL	100 CFU/ml	Research, food
**10 mL**	0.1 CFU/mL	1 CFU/mL	10 CFU/ml	Food safety
**50 mL**	0.02 CFU/mL	0.2 CFU/mL	1 CFU/ml	Sterility tests

**Table 3:** O_2_ sensor-based respirometry platforms for microbial testing.

No.	Platform	Sensor Dye and Format	Assay Substrate and Settings	Detector, Optical Readout	Application	Refs.
1.	**Bactec** **System ^1^**	Ru(dpp)_3_ (470/615 nm), solid-state coating	Coated glass vials with caps	Customized detector with incubator, intensity-based	Detection of microbial infections in blood samples	[88]
2.	**BD Biosensor** **System ^1^**	Ru-dye (470/615 nm), solid-state coating	Bottom-coated 96-WP	Standard plate reader with T control, Intensity-based	Microbial respiration (general), antimicrobials	[89]
3.	**Sensor Plates ^2.3^ e.g.,** **OxoDish**	PtPFPP^2^ (525/650 nm) or PtBP^3^ (615/760 nm), solid-state coating	Bottom-coated 24-WP	Multichannel phase detector, phase shift	Detection of microbial respiration (general)	[90]
4.	**Seahorse XF** **Analyser ^4^**	PtPFPP dye, solid- state coating on lid pins	Special 24/96-WP, plus lid with pins and sensors. Seal able microwells	Customized detector with incubator, intensity-based	Mammalian cell respiration, OCRs, multiparametric analysis of cell bio- energetics. Research apps	[73]
5.	**MitoXpress probe ^5^**	PtCP dye (390/650 nm), liquid dispensable probe	Standard 96/384- WP, other sub- strates, biochips. Oil seal.	Standard TR-F reader with T control, LT based	Detection of bacterial growth, enumeration—TVCs Analysis of food samples, antimicrobials, etc.	[78,81,82,83,84]
6.	**GreenLight 930 System ^6^**	PtBP dye (615/760 nm), solid-state coating	2 mL plastic vials with sensor dots	Benchtop carousel reader and incubator, LT based	Enumeration of bacteria, bacterial growth and inhibition assays	[82]
7.	**Sensor vial** **system**	PtBP dye (615/760 nm), solid-state coating	15/50 mL plastic vials with sensor dots	Handheld reader, LT or phase shift	Detection of bacteria in cultures, complex samples (homogenates), enumeration—TVCs	[79]
8.	**Swab vial** **system**	PtBP dye (615/760 nm), solid-state coating	15 mL vials with swab brushes and sensor dots	Handheld reader, LT or phase shift measurements	Detection of bacteria in surface, carcass, envi- ronmental swabs, enumeration—TVCs	[80]
9.	**Sensor pouches**	PtBP dye (615/760 nm), membrane insert	Sealable plastic sachets with sensor inserts	Handheld reader, LT or phase shift measurements	Detection of bacteria in various foods, enumeration —TVCs	[91]
10.	**ambr^®^ micro** **bioreactor system ^7^**	PtPFPP dye (525/650 nm), solid-state O_2_ and pH-sensitive coatings	Disposable 15 mL microbioreactors with O_2_ and pH sensors	Multichannel phase detector, phase shift	High throughput optimiza- tion of cell culturing conditions and media— bioprocessing	[92]
11.	**Fluidic** **biochips**	PtBP dye (615/760 nm), soluble NP probe ^5^	Microfluidic biochips, probe in the media	Portable benchtop reader, phase shift measurements	Mammalian cell respiration, OCRs, research apps	[75,93]
12.	**Micro-sensors ^2,3^**	PtPFPP or PtBP dye in sol–gel matrix	Sensor on tip of optical fiber probe	Portable reader, LT- based sensing— phase shift	Dipstick O_2_ and OCR probe, microbial communities, biofilms	[94]

Commercialized by: 1—Becton Dickinson; 2—Presens; 3—Pyroscience; 4—Agilent/Seahorse; 5—Agilent/Luxcel; 6—Oculer/Mocon; 7—Sartorius/Presens. Abbreviations: NP—nanoparticles; TVC—total viable counts; LT—phosphorescence lifetime; PtPFPP—Pt(II)-tetrakis-(pentafluorophenyl)porphine; PtBP—Pt(II)-benzoporphyrin; PtCP—Pt-coproporphyrin I; Ru(dpp)_3_—Ru(II)- tris(4,7-diphenyl-1,10-phenanthroline).

**Table 4:** General comparison of the different platforms for the detection and identification of viable bacterial cells (TVCs).

Parameter	Sensor Respi-Rometry	Agar Plating	Tempo BioMerieux	Soleris	ATP BL	PCR, LAMP and Alike	ELISA, Lateral Flow	IMS	Flow Cytometry	Raman, SCRS	MS
**Time to Result, h**	1–8 h	24–72 h	6–24 h	6-24 h	<1 h	6–12 h	3 h	4 h	<1 h	<1 h	1 h
**Detection of viable cells** **only**	+++	+++	+++	+++	++	+	+	++	++	+	-
**Sensitivity/LOD, CFU**	1 CFU	1 CFU	1 CFU	1 CFU	10–100	10–100	10^2^–10^5^	10–100	>1 cell	>1 cell	10^2^–10^5^
**Dynamic range**	7–8 Logs	several Logs^4^	several Logs	several Logs	4–6 Logs	3–4 Logs	2–4 Logs	3–4 Logs	3–4 Logs	unknown	unknown
**Total/Selective cell counts** **possible**	yes/yes ^1^	yes/yes	yes/yes ^3^	yes/yes	yes/yes ^3^	no/yes	no/yes	no/yes	yes/yes ^2^	no/yes ^2^	no/yes
**Sample/assay volume, mL**	0.1–50	10	1	10	0.1–1	1–2	0.1 mL	10 mL	0.1–1	0.1 mL	0.1 mL
**No of samples processed** **(batch size)**	1–50	1–20	1–100	1–20	10–100	1–10	1–10	1–10	10–20	1–10	1–20
**Sample preparation time, h**	0.3	1	0.5	0.3	0.3	3 h	1 h	1 h	0.5 h	0.5 h	1 h
**Cell lysis/enrichment/** **clarification required**	no	no	partial	partial	yes	yes	yes/no	yes/no	no	yes	yes
**Matrix effects,** **false +/--ves**	+/-	-	+	+	++	++	++	+	++	++	-
**Waste and risk of contamination**	+	+++	+	+	+	++	++	++	+	+	+
**Detector costs, $**	1–30 k	none	>30 k	1 k	5 k	5 0k	0–5 k	>5 k	>50 k	>50 k	>200 k
**Additional equipment and facilities required**	+	++	+++	+	+	+++	+	++	++	++	+++
**Assay costs, $**	1–5 $	2–0	>20	30	10	>10	3–10	10–20	>5	>10	>3

1—not demonstrated; 2—with additional steps; 3—with selective enrichment step; 4—serial dilutions required.

## Data Availability

Data is available from the authors on request.
